# A Case of Severe Hyponatremia Unmasking Borderline Heart Failure With Preserved Ejection Fraction With Right Ventricular Dysfunction

**DOI:** 10.7759/cureus.95007

**Published:** 2025-10-20

**Authors:** Urshila Ramah, Venessa Herminie, Charlie Cox, Rita Fernandez Garda

**Affiliations:** 1 Intensive Care Unit, St George’s University Hospitals NHS Foundation Trust, London, GBR

**Keywords:** diastolic heart failure, heart failure with preserved ejection fraction, hypervolemic hyponatremia, profound hyponatremia, right ventricular dysfunction

## Abstract

Hyponatremia, characterised by a low serum sodium level, is the most common electrolyte abnormality encountered in clinical practice. Its diverse etiologies and varied clinical presentations can make diagnosis and management complex, especially in the context of borderline heart failure with preserved ejection fraction (HFpEF). We report the case of a 53-year-old male who presented with slurred speech, generalised weakness, shortness of breath, and bilateral leg swelling over eight weeks. Initial investigations revealed severe hypotonic hyponatremia (sodium = 97 mmol/L), elevated urine osmolality (515 mmol/kg) and low urine sodium (<20 mmol/L). Imaging demonstrated pulmonary oedema and cardiomegaly. Liver and renal studies excluded cirrhosis and nephrotic syndrome. Echocardiography showed an estimated borderline preserved left ventricular ejection fraction, severely dilated left atrium, and impaired right ventricular function. Advanced echocardiography parameters supported a diagnosis of HFpEF based on the European Society of Cardiology scoring algorithm, namely, an increased tricuspid regurgitation velocity of 3.0 m/second, a severely elevated left atrial volume index at 73.9 mL/m² and an increased left ventricular mass index at 142 g/m². The N-terminal pro-B-type natriuretic peptide level was measured at 752 pg/mL in the context of atrial fibrillation and obesity. The patient experienced acute neurological deterioration requiring intravenous hypertonic saline, intubation, and mechanical ventilation in the intensive care unit. After excluding other potential causes of hypervolemic hyponatremia, including liver cirrhosis, nephrotic syndrome, and chronic kidney disease, this case highlights the diagnostic challenges that arise when left ventricular function and natriuretic peptide levels are not markedly abnormal. In this patient, right ventricular dysfunction and elevated atrial pressures appeared to play a central role in the development of hyponatremia. Management required a careful balance: correcting sodium levels promptly to mitigate neurological risk, while avoiding overly rapid shifts that could precipitate osmotic demyelination syndrome. Clinicians should maintain a high index of suspicion for HFpEF in patients presenting with fluid overload and hyponatremia, even when traditional heart failure markers are equivocal. A comprehensive, multidisciplinary approach, integrating biochemical data, imaging, and clinical context, is essential for accurate diagnosis and safe, effective treatment.

## Introduction

Hyponatremia is defined as a plasma sodium concentration of less than 135 mmol/L [1]. It is the most common electrolyte disorder encountered in clinical practice, with around 15-20% of hospital inpatients in the United Kingdom experiencing low sodium levels [2]. The aetiology of hyponatremia is diverse. Therefore, it is diagnostically categorised based on clinical history and volume status into hypervolemic, hypovolemic, and euvolemic subtypes [3].

In hypervolemic hyponatremia, both sodium and water are retained; however, water retention predominates, resulting in volume expansion. Potential causes include congestive heart failure, liver cirrhosis, nephrotic syndrome, and chronic kidney disease [3]. In hypovolemic hyponatremia, there is a loss of both sodium and water, such as in the use of diuretics and gastrointestinal losses, with sodium loss being proportionally greater. Euvolemic hyponatremia occurs when total body water is subtly increased without overt signs of fluid overload, often due to excessive fluid intake or syndrome of inappropriate antidiuretic hormone secretion (SIADH) [3].

The clinical manifestations of hyponatremia are highly variable, ranging from asymptomatic cases to severe symptoms such as confusion, seizures, coma, and even death. Additionally, the clinical presentation may sometimes reflect the underlying cause of the hyponatremia rather than the electrolyte disturbance itself.

Heart failure with preserved ejection fraction (HFpEF) is characterised by signs and symptoms of heart failure with a left ventricular ejection fraction (LVEF) ≥50% and elevated N-terminal pro-B-type natriuretic peptide (NT-proBNP) levels. Echocardiographic findings typically include impaired left ventricular relaxation, increased filling pressures, and left atrial enlargement [4]. These pathophysiological changes lead to elevated left atrial pressures and systemic venous congestion, resulting in renal hypoperfusion. In turn, this activates neurohormonal pathways such as the renin-angiotensin-aldosterone system and antidiuretic hormone (ADH), promoting sodium and water retention. However, water retention often exceeds sodium retention, leading to dilutional (hypervolemic) hyponatremia [4].

Hyponatremia is observed in approximately 14-25% of patients with heart failure, and in HFpEF, it has been associated with increased mortality, prolonged hospital stays, and higher readmission rates [5]. Timely and appropriate correction of serum sodium levels has been shown to improve clinical outcomes in HFpEF. Management strategies include fluid restriction, loop diuretics, angiotensin-converting enzyme inhibitors, and arginine vasopressin receptor antagonists (vaptans). However, diuretics may exacerbate sodium loss, and the use of vaptans is often limited by cost and lack of long-term outcome data [5].

The treatment of hyponatremia in HFpEF remains challenging. Current guidelines offer limited and inconsistent recommendations, largely due to the underrepresentation of HFpEF patients in clinical trials. A recent review of heart failure guidelines by the European Society of Cardiology (ESC) and American Heart Association (AHA) highlights variability in diagnostic criteria, therapeutic thresholds, and the role of vasopressin antagonists, underscoring the need for HFpEF-specific evidence [6].

We present a case of hypervolemic hyponatremia, marked by significant diagnostic challenges, likely due to borderline HFpEF.

## Case presentation

A 53-year-old male patient presented to the emergency department with slurred speech, generalised weakness, and progressively worsening shortness of breath, along with swollen legs over an eight-week period. He first noticed the swelling in his legs during a flight to Thailand and reported feeling more fatigued before the trip, which he initially attributed to increased workload. He denied any history of fever. He had no known past medical history and was not on any medications at home. He was obese, a heavy smoker, and consumed alcohol daily.

In the emergency department, he was tachypnoeic with a respiratory rate of 30 breaths per minute and had crepitations in both lung fields. His oxygen saturation was 100% on a nasal cannula at 3 L/minute. His blood pressure was 210/112 mmHg, and his heart rate was 108 beats per minute. Bilateral pitting oedema up to his knees was noted.

**Figure 1:**
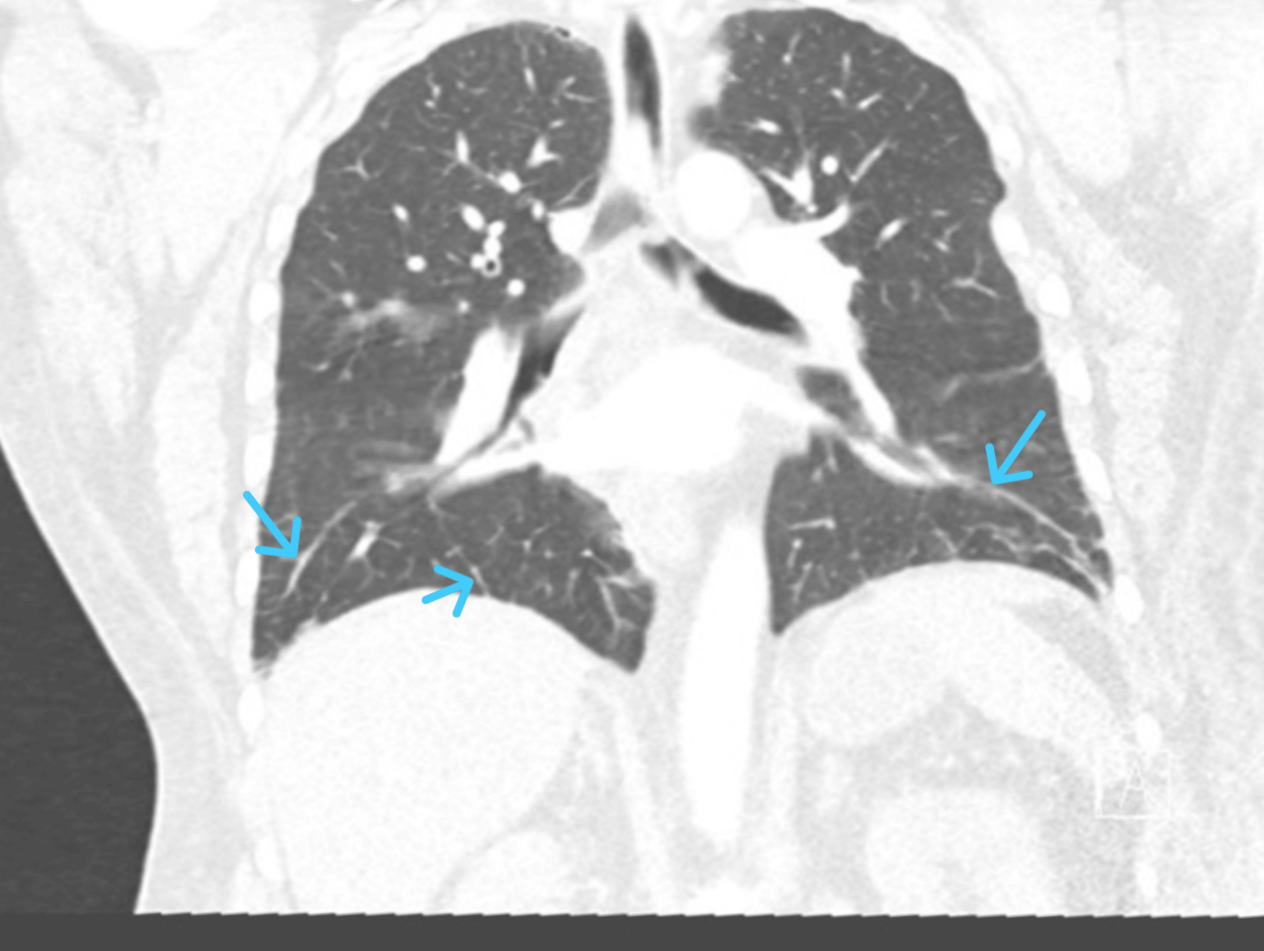
CT pulmonary angiogram on admission showing bi-basal interlobular septal thickening consistent with pulmonary edema (blue arrows).

His electrocardiogram showed a normal sinus rhythm. A venous blood gas showed a sodium level of 97 mmol/L, which was later confirmed with formal biochemistry. The initial impression was symptomatic hypervolemic hyponatraemia secondary to heart failure. He was thus treated with diuretics (intravenous (IV) furosemide 40 mg). A CT pulmonary angiogram revealed bi-basal interlobular septal thickening consistent with pulmonary oedema (Figure 1), along with cardiomegaly and flattening of the interventricular septum. A CT of the head, performed due to the neurological signs on admission, was unremarkable.

The patient was admitted to the intensive care unit (ICU) due to profound hyponatremia with associated neurological symptoms. Management focused on cautious sodium correction to prevent osmotic demyelination syndrome. Fluid intake was restricted to 1 L/day, and two boluses of 1.8% hypertonic saline were administered at a dose of 2 mL/kg each. The therapeutic goal was to increase serum sodium by no more than 8 mmol/L per 24 hours. Regular arterial blood gas analyses were performed following each hypertonic saline bolus to monitor serum sodium levels, ensuring safe and controlled correction.

His echocardiogram showed dilated ventricles, mildly impaired left ventricular systolic function (estimated LVEF = 45-50%), a severely dilated left atrium (left atrial volume index = 73.94 mL/m²), evidence of elevated left atrial pressures, impaired right ventricular systolic function with some evidence of raised pulmonary pressures (maximum tricuspid regurgitation velocity = 3 m/second, tricuspid annular plane systolic excursion = 2 cm), right atrial dilation (right artial area index = 14.29 cm²/m²), and impaired right ventricular radial function.

Further tests allowed us to classify the hypervolemic hyponatremia as hypotonic with normal renal function and without renal loss of sodium (Table 1). Thus, we could rule out SIADH. NT-proBNP levels were within the normal range for his age; however, interpretation was confounded by the patient’s obesity, which is known to suppress natriuretic peptide concentrations and may mask underlying cardiac dysfunction.

**Table 1:** Further investigations for the patient’s hyponatremia. NT-ProBNP: N-terminal pro-B-type natriuretic peptide

Test	Value	Reference range
Plasma osmolality	216	275–295 mmol/kg
Blood urea	1.0	2.5–7.5 mmol/L
Blood creatinine	53	60–106 µmol/L
Blood sodium	108	133–146 mmol/L
NT-ProBNP level	752 ng/L	0–899 ng/L
Urine osmolality	515	100–1,400 mmol/kg
Urine sodium level	<20	>20 mmol/L – renal loss

**Figure 2:**
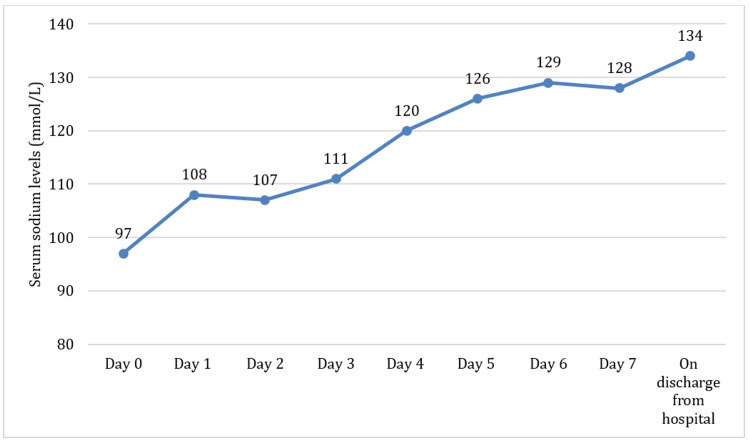
Trends in serum sodium levels from admission to discharge.

Twenty-four hours into his ICU admission, the patient had a profound reduction in his level of consciousness, leading to an inability to protect his airway. This was a rapid deterioration in his condition, leading to an eventual hypoxic cardiac arrest. One cycle of cardiopulmonary resuscitation was performed, after which return of spontaneous circulation was achieved. He was subsequently intubated and mechanically ventilated. At that time, his sodium levels were 111 mmol/L (Figure 2). Over the next 48 hours, his sodium levels improved (from 111 mmol/L to 126 mmol/L) after administration of further boluses of hypertonic saline and continued fluid restriction. His neurology improved considerably, and he was extubated.

The underlying cause of hyponatremia in this case remained uncertain, as the echocardiographic findings did not fully align with the severity of hyponatremia observed on admission. Moreover, the estimated ejection fraction fell into a borderline range, making it difficult to definitively classify the patient within a specific heart failure phenotype. Both the heart failure team and endocrinology teams were consulted as part of multi-disciplinary discussions.

An ultrasound of his liver revealed hepatic steatosis without signs of cirrhosis. The liver enzymes were mildly elevated (alanine aminotransferase = 53 U/L; normal value = 0-41 U/L), and serum albumin was 37 g/L (normal value = 35-50 g/L). A urine albumin-to-creatinine ratio was abnormal (50.5 mg/mmol; normal value <3 mg/mmol), and urine albumin levels were elevated (869 mg/L; normal value <20 mg/L), which was attributed to his critical illness. His thyroid function tests, blood glucose levels, serum potassium, serum cortisol levels, and lipid profile were all normal, excluding adrenal insufficiency, thyroid disorders, and hyperlipidemia as other possible causes of hyponatremia.

Other significant events during his hospitalisation included hospital-acquired pneumonia, for which he was treated with co-amoxiclav, respiratory acidosis (likely due to obstructive sleep apnoea or obesity hypoventilation syndrome), and new-onset atrial fibrillation (AF).

After extubation and improvement of his sodium levels (Figure 2), he was transferred to the ward and discharged home a few days later with follow-up appointments in the acute medicine clinic and the respiratory team. His serum sodium level at discharge was 134 mmol/L. Discharge medications included apixaban for the AF, bisoprolol, furosemide, and a fluticasone/vilanterol inhaler.

## Discussion

This case represents a diagnostically challenging presentation of hyponatremia in a middle-aged man. The patient developed progressive features of fluid overload over an eight-week period and subsequently manifested neurological symptoms, most likely secondary to severe hyponatremia. Integration of the clinical presentation with laboratory investigations, imaging studies, and echocardiography suggested a diagnosis of hypervolemic hyponatremia. Liver ultrasound excluded cirrhosis, while preserved renal function and normal serum albumin made nephrotic syndrome unlikely. CT pulmonary angiogram revealed pulmonary oedema and cardiomegaly, and transthoracic echocardiography demonstrated an LVEF of 45-50%, impaired right ventricular systolic function, and dilated left atrium with elevated filling pressures. Taken together, the results indicated that heart failure was the most probable cause of hyponatremia.

Hyponatremia is the most common electrolyte abnormality in patients with heart failure, with a reported prevalence of 20-25% [7,8]. This is explained by impaired renal free water excretion secondary to elevated ADH levels in heart failure [9]. Furthermore, lower sodium levels are associated with more severe left ventricular dysfunction and higher NT-proBNP levels [10].

Our patient had an estimated LVEF of 45-50%, placing him in a borderline range between preserved and mildly reduced systolic function. NT-proBNP levels appeared normal for his age; however, we believe this was falsely low due to the patient’s obesity, which is known to suppress natriuretic peptide concentrations. Additionally, echocardiographic assessment was technically challenging due to the patient’s body habitus, and the LVEF value was an approximation rather than a definitive measurement. These factors collectively complicated our diagnostic approach and limited our ability to confidently classify the heart failure phenotype. He did, on the other hand, have evidence of right ventricular dysfunction. Lee et al. described an association between right ventricular dysfunction and hyponatremia in a nested case-control study of the Korean Heart Failure Registry [11]. Right ventricular dysfunction causes systemic venous congestion and reduced renal perfusion, triggering ADH release and water retention. This neurohormonal activation leads to dilutional hyponatremia, even with preserved left ventricular function.

Half of current heart failure admissions involve heart failure with HFpEF, though diagnosis remains challenging [12]. HFpEF patients typically present with symptoms and signs of heart failure despite a normal LVEF [13]. Although mortality is lower in this group, they experience similar degrees of symptom burden, readmission risk, and functional limitation compared to patients with reduced ejection fraction [14]. LVEF, being a measure of systolic function, does not always correlate with overall heart failure symptom severity.

In our patient, accurate assessment of diastolic function was significantly limited by the presence of AF. The rhythm irregularity precluded reliable measurement of mitral annular early diastolic velocity (e′), which is essential for calculating the E/e′ ratio, a key index of left ventricular filling pressure [15]. Consequently, we were unable to apply the H₂FPEF score, which incorporates E/e′ as a central component. This underscores the diagnostic challenge posed by AF in evaluating HFpEF, where surrogate markers such as left atrial volume index, tricuspid regurgitation velocity, and left ventricular mass index become critical in supporting the diagnosis.

Applying the ESC 2019 diagnostic algorithm for HFpEF [12], our patient scored 6 points, supporting a diagnosis of HFpEF: functional domain: tricuspid regurgitation velocity of 3.0 m/second (major criterion, 2 points); morphological domain: severely elevated left atrial volume index (73.9 mL/m², major criterion, 2 points); elevated left ventricular mass index (142 g/m², minor criterion; and biomarker domain: NT-proBNP level of 752 pg/mL in the context of atrial fibrillation (major criterion threshold: >365 pg/mL in AF, 2 points). Although assessment of diastolic function was limited by rhythm irregularity, these surrogate markers provided substantial evidence supporting the diagnosis.

Obesity significantly influences both the interpretation of BNP levels and the pathophysiology of HFpEF. In obese individuals, BNP concentrations are often deceptively low due to increased clearance and reduced synthesis of natriuretic peptides, which can mask the diagnosis of HFpEF despite elevated filling pressures [12]. Obesity contributes to HFpEF through mechanisms such as systemic inflammation, epicardial fat deposition, and altered myocardial energetics. These changes promote left ventricular stiffness, diastolic dysfunction, and pulmonary vascular remodelling, resulting in a distinct HFpEF phenotype with biventricular involvement and preserved ejection fraction [16]. Consequently, clinicians must interpret BNP cautiously in obese patients and rely on structural and functional echocardiographic markers to support HFpEF diagnosis.

While beer potomania can also present with hypotonic hyponatremia, patients are typically hypovolemic or euvolemic and urine osmolality is typically low [17]. These findings were not consistent with our case. Urine analysis showed a raised albumin-to-creatinine ratio and elevated urine albumin. Given the absence of hematuria, preserved serum albumin, and normal renal function, we did not suspect intrinsic renal disease or nephrotic syndrome as causes of hyponatremia in this case. The proteinuria may have been transient, secondary to acute illness or hypertensive crisis on admission [18].

As previously mentioned, this case highlights the delicate balance between urgency and caution in managing symptomatic hyponatremia and the need for frequent monitoring of sodium levels during the acute phase. While urgent hypertonic saline therapy was needed, correction was limited by the risk of osmotic demyelination syndrome (ODS), a complication of rapid overcorrection of serum sodium, which is also more common in severe hyponatremia. This posed a therapeutic dilemma: rapid correction to prevent further neurological decline versus the potential for irreversible ODS.

Guidelines advise limiting correction to ≤8 mmol/L over 24 hours when sodium is <120 mmol/L or when additional ODS risk factors are present (e.g., alcohol use disorder, malnutrition, advanced liver disease) [19]. For patients with severe symptoms, the European Clinical Practice Guidelines and the US/Irish expert panel recommend hypertonic saline boluses aiming for a rise in sodium of 4-6 mmol/L over the first few hours [20]. In our patient, we adhered to these safety parameters by ensuring that the serum sodium did not increase by more than 8 mmol/L over any 24-hour period. Serial sodium measurements were performed after each hypertonic saline bolus to guide therapy and minimise the risk of overcorrection.

## Conclusions

This case highlights the diagnostic approach to hyponatremia in the setting of clinical hypervolemia and reinforces the necessity of a structured, multidisciplinary approach to uncover its aetiology. Complexities in this case included a falsely low NT-proBNP due to obesity and estimation of a borderline LVEF, causing diagnostic difficulty to classify into one heart failure phenotype. However, in the presence of right ventricular dysfunction, pulmonary congestion, and elevated left atrial pressures, we still pursued heart failure as the most plausible cause. Application of the ESC diagnostic algorithm supported a diagnosis of HFpEF, emphasising the importance of considering this entity even when natriuretic peptide levels and systolic function do not appear to be grossly abnormal. This case also underscores the diagnostic limitations posed by AF, which precluded accurate diastolic assessment via E/e′ ratio and limited the use of other scoring systems such as H₂FPEF. From a management perspective, the case illustrates the delicate balance between prompt correction of symptomatic hyponatremia and the risk of ODS, highlighting the need for vigilant monitoring and individualised sodium correction strategies. Clinically, this case supports the integration of echocardiographic surrogates and clinical context when standard markers are inconclusive. Future research should focus on refining diagnostic algorithms for HFpEF in patients with arrhythmias and obesity, and exploring tailored strategies for managing hyponatremia in this complex population.
